# Prostaglandin-E2 receptor-4 stimulant rescues cardiac malfunction during myocarditis and protects the heart from adverse ventricular remodeling after myocarditis

**DOI:** 10.1038/s41598-021-99930-5

**Published:** 2021-10-26

**Authors:** Akira Takakuma, Mototsugu Nishii, Alan Valaperti, Haruto Hiraga, Ryo Saji, Kazuya Sakai, Reo Matsumura, Yasuo Miyata, Nozomu Oba, Fumiya Nunose, Fumihiro Ogawa, Kouichi Tamura, Ichiro Takeuchi

**Affiliations:** 1 Department of Emergency Medicine, Yokohama City University Graduate School of Medicine, 3-9 Fukuura, Kanazawa-ku, Yokohama, Japan 236-0004; 2 Department of Immunology, University Hospital Zurich, Schmelzbergstrasse 26, CH-8091 Zurich, Switzerland; 3 Department of Medical Science and Cardiorenal Medicine, Yokohama City University Graduate School of Medicine, 3-9 Fukuura, Kanazawa-ku, Yokohama, Japan 236-0004,

**Keywords:** Molecular biology, Cardiology

## Abstract

Cardioprotective effect of prostaglandin-E2 receptor-4 (EP4) stimulation on the ischemic heart has been demonstrated. Its effect on the heart affected by myocarditis, however, remains uncertain. In this study, we investigated therapeutic effect of EP4 stimulant using a mouse model of autoimmune myocarditis (EAM) that progresses to dilated cardiomyopathy (DCM). EP4 was present in the hearts of EAM mice. Treatment with EP4 agonist (ONO-0260164: 20 mg/kg/day) improved an impaired left ventricular (LV) contractility and reduction of blood pressure on day 21, a peak myocardial inflammation. Alternatively, DCM phenotype, characterized by LV dilation, LV systolic dysfunction, and collagen deposition, was observed on day 56, along with activation of matrix metalloproteinase (MMP)-2 critical for myocardial extracellular matrix disruption, indicating an important molecular mechanism underlying adverse ventricular remodeling after myocarditis. Continued treatment with ONO-0260164 alleviated the DCM phenotype, but this effect was counteracted by its combination with a EP4 antagonist. Moreover, ONO-0260164 inhibited in vivo proteolytic activity of MMP-2 in association with up-regulation of tissue inhibitor of metalloproteinase (TIMP)-3. EP4 stimulant may be a promising and novel therapeutic agent that rescues cardiac malfunction during myocarditis and prevents adverse ventricular remodeling after myocarditis by promoting the TIMP-3/MMP-2 axis.

## Introduction

Myocarditis is an inflammatory heart disease characterized by broad range of clinical course from cardiac malfunction requiring inotropic drugs and/or mechanical circulatory support to dilated cardiomyopathy (DCM) requiring heart transplantation^1,2^. Pharmacological treatments for inflammatory heart disease, however, have yet to be established.

Apart from chronic viral infection^3^, immunohistochemical evidence and the presence of autoantibodies against myocardial structure have showed that autoimmune response, which follows the myocardial damage provoked by the initial viral infection, plays a crucial role in the pathogenesis of myocarditis and subsequent DCM^4,5^. DCM pathology is also characterized by adverse ventricular remodeling that manifests as left ventricular (LV) dilation, impaired LV contractility, and fibrosis. Disruption of myocardial extracellular matrix (ECM), that provides myocardial wall mechanics, by matrix metalloproteinases (MMPs) activation^6^ critically provokes adverse ventricular remodeling^7^. Cardiac fibrosis characterized by accumulation of collagen deposit in the myocardial interstitium^8^ also results in profound impairment of LV systolic and diastolic function via disrupting the coordination of myocardial excitation–contraction coupling in both systole and diastole^9,10^.

Prostaglandin (PG) E2 (PGE2) has been implicated in the pathogenesis of inflammatory diseases through four specific receptors (EP 1–4 receptors)^11^. Particularly, functional and morphological analyses using genetic modification and selective agonist and antagonist have shown that EP4 receptor stimulation protects the heart from cardiac rupture and improves cardiac function after ischemia–reperfusion injury^12–14^ and further alleviates cardiac fibrosis in pressure-overloaded heart^15^. Its effect on the heart affected by myocarditis, however, remains elusive.

The BALB/c mouse model of experimental autoimmune myocarditis (EAM) allows to study not only autoimmune mechanisms, but also transition from myocarditis to DCM^16^. In the present study, we investigated the molecular mechanisms underlying adverse ventricular remodeling caused by myocarditis and related therapeutic effect of EP4 receptor stimulant using a mouse EAM model and showed for the first time that EP4 stimulant protects the heart from adverse ventricular remodeling after myocarditis by controlling myocardial ECM metabolism.

## Results

### EP4 receptor expression in EAM

Immunoblot in bulk heart tissue showed that EP4 receptor is present in the hearts of EAM mice. The expression of EP4 increased rapidly from day 14 to day 21 and persisted thereafter (Supplementary Fig. 1A). Moreover, immunostaining at 21 days after immunization, when myocardial inflammation was at its peak, showed expression of EP4 receptor in infiltrating cells and myocytes around the inflamed site on the endocardium and epicardium-sided LV wall, rather than in whole myocardium (Supplementary Fig. 1B).

### Effects of a EP4 receptor selective agonist in healthy mice

To identify optimal dose of ONO-0260164, a EP4 receptor selective agonist for in vivo study, systolic blood pressure (BP), body weight (BW), and echocardiographic parameters were evaluated in healthy mice treated with ONO-0260164. The BP was significantly decreased 1 or 2 h after a single administration of ONO-0260164 (20 or 50 mg/kg) compared with before the administration, although the heart rate (HR) did not change between before and after the administration (Supplementary Fig. 2A). Conversely, daily administration of ONO-0260164 (20 or 50 mg/kg/day) significantly reduced HR on days 21 and 49 but had no effect on BP (Supplementary Fig. 2B). Alternatively, the BW was significantly increased in the 50 mg/kg/day group of ONO-0260164 compared to the vehicle only group, but not in 2 or 20 mg/kg/day group. The change in BW from day 21 to day 49 also followed the same manner (Supplementary Fig. 2C).

Daily administration of ONO-0260164 did not affect echocardiographic parameters on day 21, including LV end-diastolic dimension (LVDd), LV end-systolic dimension (LVDs), interventricular septum diastolic thickness (IVSd), LV posterior wall diastolic thickness (PWd), LV fractional shortening (FS), and LV mass index (LVMI) (Supplementary Fig. 3A). However, LVMI as well as IVSd and PWd on day 49 was significantly increased in the 50 mg/kg/day group of ONO-0260164 compared to the vehicle only group, but not in the 20 mg/kg/day group of ONO-0260164 (Supplementary Fig. 3B). Therefore, we decided to use 20 mg/kg/day of ONO-0260164 for in vivo investigation.

### Immunomodulation by EP4 receptor signaling in acute EAM

**Figure 1:**
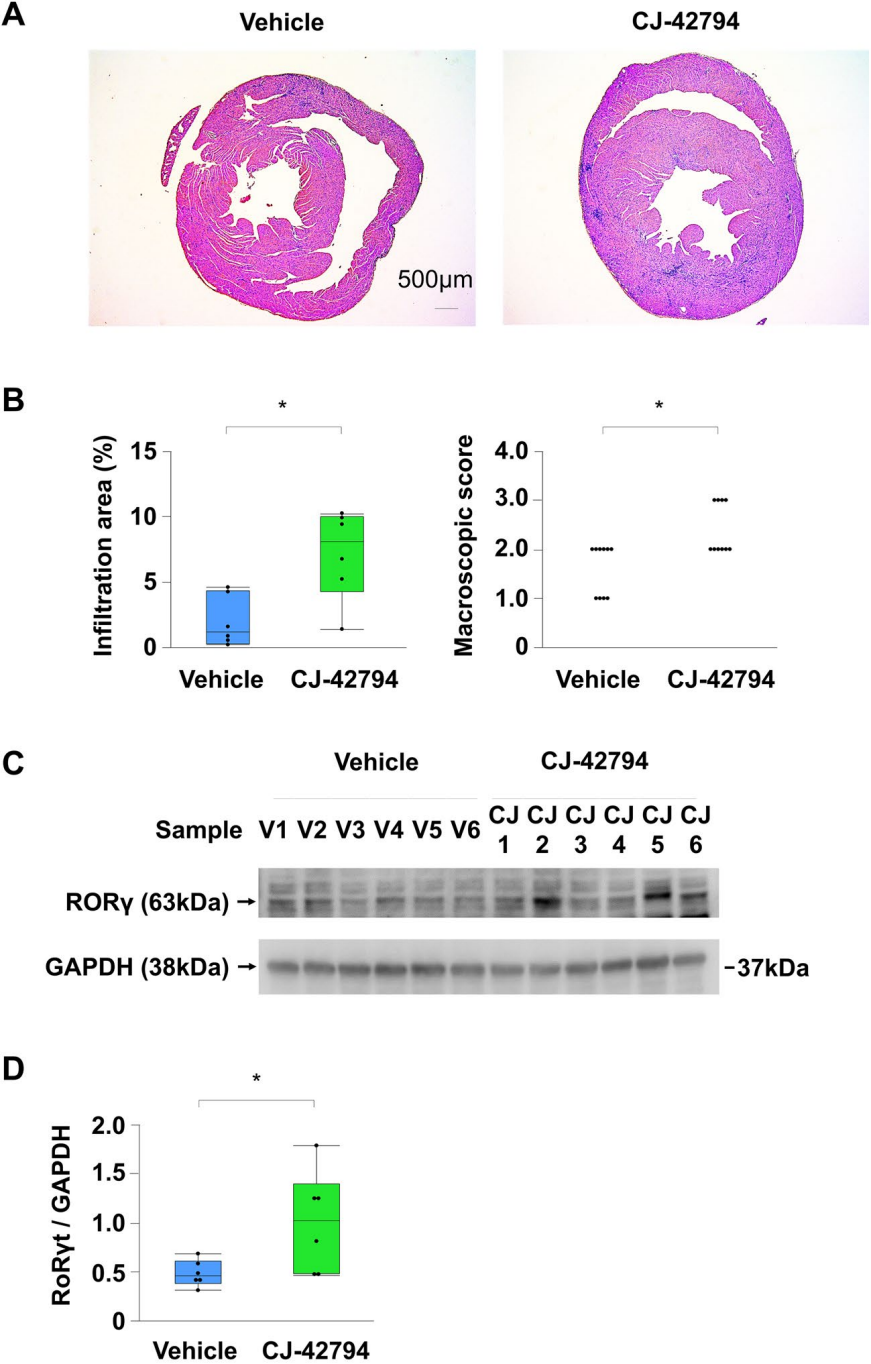
Myocardial inflammation in the experimental autoimmune myocarditis (EAM). (**A**) Representative Hematoxylin–Eosin staining and (**B**) statistical analyses of inflamed area (n = 6/each) and macroscopic score (n = 10/each) in ventricular cross-sections and hearts, respectively on day 21 obtained from EAM treated daily with vehicle or selective prostaglandin E2 receptor 4 (EP4) antagonist (CJ-42794) from day 14 to day 21. Scale bars: 500 μm. Inflamed area was calculated by affected area/ventricular area × 100%. (**C**) Western blot (cropped gel) and (**D**) its densitometric analysis (n = 6/each) of the EAM inducible Th17-specific master transcription factor, retinoic acid receptor-related orphan nuclear receptor (ROR γt) on day 21 hearts from EAM mice treated with vehicle or CJ-42794. Full-length blots of ROR γt and GAPDH are presented in Supplementary Fig. 4A. *P < 0.05 (vs. vehicle) calculated with nonparametric 2-tailed Mann–Whitney *U* test.

In contrast to a previous report showing the anti-inflammatory effect of a selective EP4 receptor agonist on acute EAM^17^, daily treatment with CJ-42794, a selective EP4 antagonist, from day 14 to day 21, significantly exacerbated myocardial inflammation on day 21 compared to vehicle alone (inflamed area: 7.2 ± 1.4% vs. 2.0 ± 0.8%, *P* = 0.0202; macroscopic score: 2.4 ± 0.2 vs. 1.6 ± 0.2, *P* = 0.0067; respectively) (Fig. 1A,B). Consistently, cardiac protein expression of the EAM inducible Th17-specific master transcription factor, retinoic acid receptor-related orphan nuclear receptor (ROR γt)^18^ on day 21 was significantly increased in CJ-42794-treated EAM compared with in vehicle-treated EAM (The ratio of ROR γt to GAPDH: 0.99 ± 0.21 vs. 0.48 ± 0.05, *P* = 0.0453, respectively) (Fig. 1C,D).

These observations suggest that EP4 receptor stimulation modulates the development of myocardial inflammation in the EAM model.

### Cardiac malfunction in acute phase of EAM mice and its control by EP4 receptor stimulation

To evaluate the effects of EP4 receptor stimulation on cardiac function and hemodynamics during acute myocarditis, echocardiography and blood pressure measurements were performed before and after administration, i.e., on days 14 and 21, respectively. On day 14, EAM mice showed impaired LV contractility with significantly lower LVFS and higher LVDs compared to non-EAM mice, but had no LV dilation, as there was no significant difference of LVDd between EAM and non-EAM (Supplementary Table 1). On the other hand, on day 21, EAM mice showed not only impairment of LV contractility but also LV dilation, as indicated by significantly lower LVFS and higher LVDs and LVDd than non-EAM mice. Conversely, daily administration of ONO-0260164 to EAM mice resulted in significantly higher LVFS and lower LV size compared with vehicle-only administration to EAM mice, suggesting that ONO-0260164 positively affects cardiac function during acute myocarditis (Table 1 and Supplementary Video 1). To evaluate if ONO-0260164 exerts its beneficial effect via the EP4 receptor, we next tested the effect of ONO-0260164 in combination with CJ-42794, a selective EP4 receptor antagonist. Consistently, on day 21, EAM mice had significantly lower LVFS and higher LVDs and LVDd compared to non-EAM mice. Moreover, daily administration of ONO-0260164 to EAM mice resulted in significantly higher LVFS and lower LV size than when EAM mice were treated with vehicle alone. However, these differences became unclear when combined with CJ-42794 (Table 2).

**Table 1:** Echocardiographic data on day 21 in experimental autoimmune myocarditis (EAM) mice.

Parameters	G1 (n = 5)	G2 (n = 8)	G3 (n = 7)	*P* values		
				ANOVA	G1 versus G2	G2 versus G3
LVFS (%)	74.1 ± 1.1 [71.1–77.2]	39.5 ± 4.1 [29.8–49.3]	68.8 ± 3.2 [60.9–76.7]	< 0.0001	< 0.0001	< 0.0001
LVDs (mm)	0.67 ± 0.05 [0.54–0.80]	1.92 ± 0.28 [1.27–2.57]	0.69 ± 0.08 [0.49–0.89]	0.0006	0.0006	0.0007
LVDd (mm)	2.57 ± 0.04 [2.46–2.67]	3.06 ± 0.24 [2.50–3.61]	2.19 ± 0.11 [1.92–2.47]	0.0161	0.0373	0.0002
HR (bpm)	675.6 ± 15.7 [632.0–719.3]	687.1 ± 14.9 [651.8–722.4]	669.5 ± 18.2 [625.1–714.0]	1.4574		
BP (mmHg)	117.4 ± 3.1 [108.9–125.9]	92.9 ± 2.7 [86.5–99.4]	113.5 ± 2.6 [107.5–119.4]	0.0102	0.0303	0.003

Data are mean ± SEM [95% confidence interval]. Echocardiographic findings on day 21 in non-EAM mice daily treated with vehicle only (G1: n = 5) or in EAM mice treated with vehicle (G2: n = 8) or selective prostaglandin E2 receptor 4 agonist (ONO-0260164) (G3: n = 7) from day 14 to day 21.

P values were calculated with 2-way ANOVA followed by the Bonferroni-Dunn post hoc testing method.

*LVFS* left ventricular fractional shortening, *LVDs* LV end-systolic dimension, *LVDd* LV end-diastolic dimension, *HR* heart rate, *BP* systolic blood pressure.

**Table 2:** Echocardiographic data on day 21 in experimental autoimmune myocarditis (EAM) mice.

Parameters	G1 (n = 5)	G2 (n = 7)	G3 (n = 8)	G4 (n = 7)	*P* values			
					ANOVA	G1 vs. G2	G2 vs. G3	G2 vs. G4
LVFS (%)	76.5 ± 0.8 [74.4–78.7]	46.6 ± 4.8 [34.8–58.4]	62.2 ± 3.5 [53.8–70.5]	49.7 ± 5.1 [37.2–62.2]	0.0007	0.0003	0.0491	0.9537
LVDs (mm)	0.53 ± 0.04 [0.41–0.65]	1.57 ± 0.19 [1.12–2.03]	0.90 ± 0.09 [0.69–1.11]	1.54 ± 0.25 [0.94–2.15]	0.0011	0.0028	0.034	0.9993
LVDd (mm)	2.49 ± 0.10 [2.20–2.78]	2.91 ± 0.08 [2.70–3.11]	2.39 ± 0.05 [2.26–2.51]	2.92 ± 0.19 [2.45–3.40]	0.0106	0.1294	0.018	0.9997
HR (bpm)	675.6 ± 15.7 [632.0–719.3]	703.4 ± 16.5 [663.1–743.7]	684.5 ± 16.5 [642.1–726.9]	671.4 ± 16.6 [630.8–712.1]	1.0224			

Data are mean ± SEM [95% confidence interval]. Echocardiographic findings on day 21 in non-EAM mice daily treated with vehicle only (G1: n = 5) or in EAM mice daily treated with vehicle (G2: n = 7), selective prostaglandin E2 receptor 4 (EP4) agonist (ONO-0260164) (G3: n = 8), or both ONO-0260164 and selective EP4 antagonist (CJ-42794) (n = 7) from day 14 to day 21.

P values were calculated with 2-way ANOVA followed by the Bonferroni-Dunn post hoc testing method.

*LVFS* left ventricular fractional shortening, *LVDs* LV end-systolic dimension, *LVDd* LV end-diastolic dimension, *HR* heart rate.

On day 14, EAM mice showed significantly lower BP and HR compared to non-EAM mice. Moreover, the significant reduction of BP, but not HR, was also observed on day 21. However, daily administration of ONO-0260164 to EAM mice resulted in significantly higher BP on day 21 compared with vehicle-only administration to EAM mice (Supplementary Table 1 and Table 1).

These results suggest that treatment with ONO-0260164 improves cardiac malfunction during myocarditis via EP4 receptor stimulation, with positively affecting the BP.

### DCM phenotype in late phase of EAM mice and its prevention by EP4 receptor stimulation

DCM phenotype characterized by impairment of LV contractility and LV dilation was evaluated with echocardiography on day 56. DCM phenotype persisted in the late phase of EAM, as indicated by significantly lower LVFS, IVSs, and PWs and higher LVDs and LVDd compared to non-EAM mice. However, daily administration of ONO-0260164 to EAM mice resulted in significantly higher LVFS, IVSs, and PWs and lower LV size compared with vehicle-only administration to EAM mice. Moreover, these parameters did not show significant differences between ONO-0260164-treated EAM and non-EAM, suggesting that continued treatment with ONO-0260164 completely suppressed adverse LV remodeling after myocarditis (Table 3 and Supplementary Video 2). To evaluate if ONO-0260164 exerts its cardioprotective effect via the EP4 receptor, we next tested the effect of ONO-0260164 in combination with CJ-42794, a selective EP4 receptor antagonist. Consistently, on day 56, EAM mice persistently showed significantly lower LVFS and IVSs and higher LVDs and LVDd compared to non-EAM mice, while daily administration of ONO-0260164 to EAM mice resulted in significantly higher LVFS and IVSs and lower LV size than when EAM mice were treated with vehicle alone. However, daily administration of ONO-0260164 did not show those significant differences when combined with CJ-42794 (Table 4).

**Table 3:** Echocardiographic data on day 56 in experimental autoimmune myocarditis (EAM) mice.

Parameters	G1 (n = 5)	G2 (n = 8)	G3 (n = 7)	*P* values			
				ANOVA	G1 versus G2	G2 versus G3	G1 versus G3
LVFS (%)	72.4 ± 2.4 [65.8–79.1]	37.4 ± 5.2 [25.3–50.0]	62.3 ± 4.2 [52.0–72.6]	0.0003	0.0002	0.0018	0.3405
LVDs (mm)	0.68 ± 0.03 [0.59–0.77]	2.03 ± 0.40 [1.08–2.98]	0.95 ± 0.19 [0.49–1.41]	0.0251	0.0018	0.0023	0.8896
IVSs (mm)	2.05 ± 0.04 [1.94–2.16]	1.55 ± 0.14 [1.22–1.87]	2.01 ± 0.08 [1.81–2.21]	0.0118	0.0008	0.0006	1.5998
PWs (mm)	1.66 ± 0.04 [1.55–1.76]	1.37 ± 0.06 [1.22–1.52]	1.60 ± 0.06 [1.44–1.76]	0.0185	0.0008	0.0019	0.8301
LVDd (mm)	2.59 ± 0.08 [2.36–2.81]	3.17 ± 0.29 [2.47–3.87]	2.28 ± 0.07 [2.11–2.45]	0.0389	0.0368	0.0008	0.4137
HR (bpm)	690.3 ± 24.0 [623.6–757.0]	646.9 ± 15.2 [611.0–682.7]	663.8 ± 21.3 [611.7–715.8]	0.6895			
BP (mmHg)	116.2 ± 2.2 [110.1–122.3]	122.5 ± 2.4 [116.9–128.0]	120.2 ± 3.3 [112.7–127.8]	0.8914			

Data are mean ± SEM [95% confidence interval]. Echocardiographic findings on day 56 in non-EAM mice treated daily with vehicle only (G1: n = 5) or in EAM mice treated daily with vehicle (G2: n = 8) or selective prostaglandin E2 receptor 4 agonist (ONO-0260164) (G3: n = 7) from day 14 to day 56.

P values were calculated with 2-way ANOVA followed by the Bonferroni-Dunn post hoc testing method.

*LVFS* left ventricular fractional shortening, *LVDs* LV end-systolic dimension, *IVSs* interventricular septum systolic thickness, *PWs* LV posterior wall systolic thickness, *LVDd* LV end-diastolic dimension, *HR* heart rate, *BP* systolic blood pressure.

**Table 4:** Echocardiographic data on day 56 in experimental autoimmune myocarditis (EAM) mice.

Parameters	G1 (n = 5)	G2 (n = 7)	G3 (n = 8)	G4 (n = 7)	*P* values			
					ANOVA	G1 versus G2	G2 versus G3	G2 versus G4
LVFS (%)	68.0 ± 2.3 [61.6–74.5]	47.5 ± 4.0 [37.8–57.2]	64.8 ± 2.9 [57.9–71.7]	52.0 ± 5.9 [37.0–67.1]	0.0078	0.0115	0.0166	0.8523
LVDs (mm)	0.78 ± 0.05 [0.62–0.93]	1.57 ± 0.15 [1.19–1.95]	0.84 ± 0.07 [0.67–1.00]	1.53 ± 0.29 [0.78–2.27]	0.0042	0.0181	0.0132	0.9979
IVSs (mm)	2.04 ± 0.03 [1.95–2.14]	1.62 ± 0.13 [1.29–1.94]	1.98 ± 0.07 [1.81–2.14]	1.72 ± 0.08 [1.50–1.93]	0.022	0.0267	0.0349	0.8616
LVDd (mm)	2.60 ± 0.02 [2.55–2.66]	3.01 ± 0.05 [2.88–3.15]	2.39 ± 0.07 [2.23–2.55]	3.13 ± 0.18 [2.66–3.60]	< 0.0001	0.0021	0.0004	0.8302
HR (bpm)	697.3 ± 20.6 [640.1–754.6]	694.1 ± 21.3 [642.0–746.3]	649.0 ± 19.5 [603.0–695.0]	682.6 ± 18.2 [635.7–729.4]	0.5891			

Data are mean ± SEM [95% confidence interval]. Echocardiographic findings on day 56 in non-EAM mice treated daily with vehicle only (G1: n = 5) or in EAM mice treated daily with vehicle (G2: n = 7), selective prostaglandin E2 receptor 4 (EP4) agonist (ONO-0260164) (G3: n = 8), or both ONO-0260164 and selective EP4 antagonist (CJ-42794) (G4: n = 7) from day 14 to day 56.

P values were calculated with 2-way ANOVA followed by the Bonferroni-Dunn post hoc testing method.

*LVFS* Left ventricular fractional shortening, *LVDs* LV end-systolic dimension, *IVSs* Interventricular septum systolic thickness, *LVDd* LV end-diastolic dimension, *HR* Heart rate.

On day 56, the late EAM mice had no significant reduction of BP compared to non-EAM mice, and continued treatment with ONO-0260164 did not affect the BP in the late EAM mice (Table 3).

Collectively, continued treatment with ONO-0260164 prevented the development of DCM phenotype after myocarditis via EP4 receptor stimulation, without affecting blood pressure.

### Myocardial collagen deposition in late phase of EAM mice and its control by EP4 receptor stimulation

**Figure 2:**
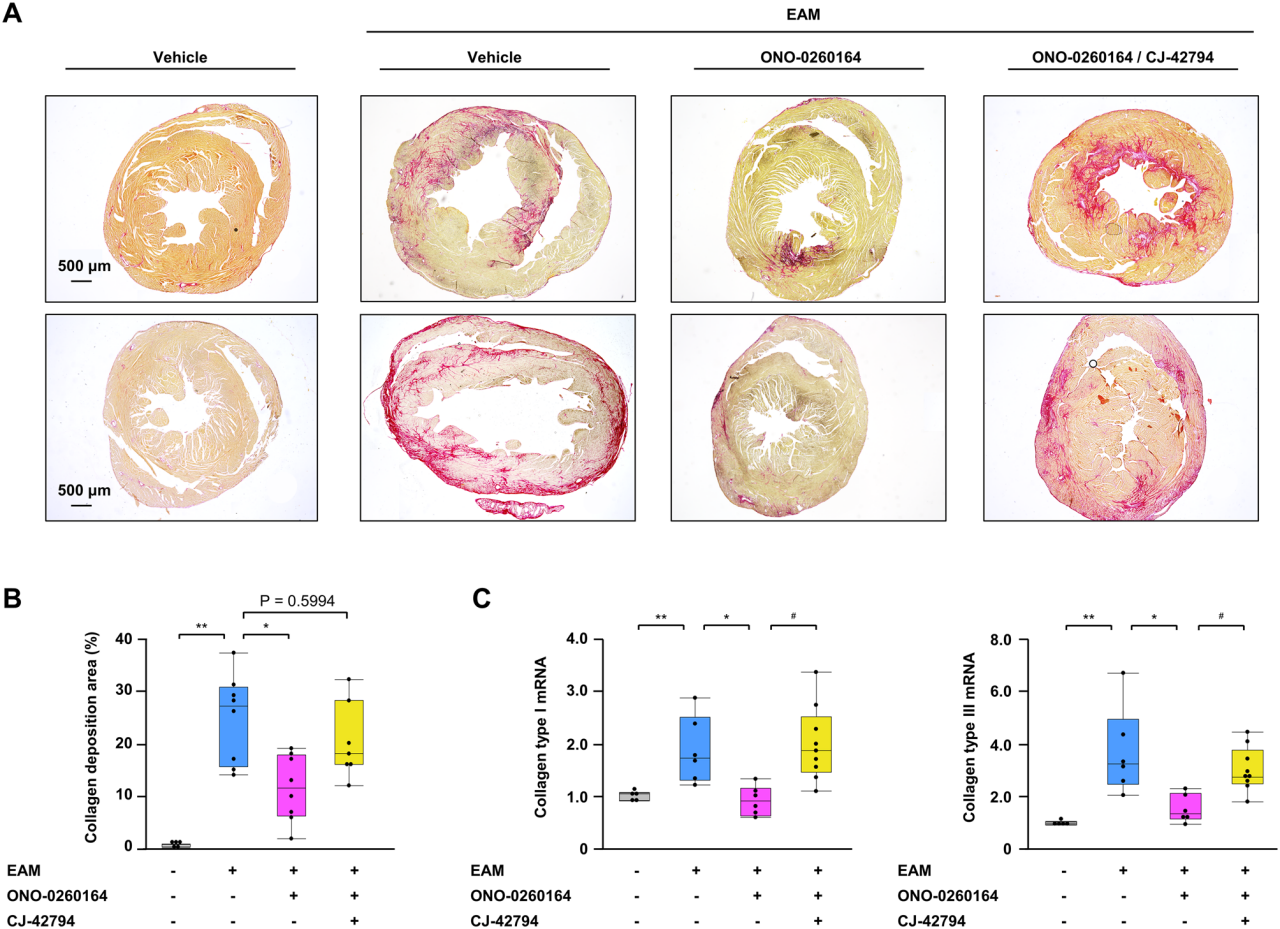
Myocardial collagen deposition in the experimental autoimmune myocarditis (EAM) mice. (**A**) Representative picrosirius red staining and (**B**) statistical analyses of collagen deposition area in ventricular cross-sections on day 56 obtained from non-EAM mice treated daily with vehicle (n = 5) or from EAM mice treated daily with vehicle (n = 8), selective prostaglandin E2 receptor 4 (EP4) agonist (ONO-0260164) (n = 8), or both ONO-0260164 and selective EP4 antagonist (CJ-42794) (n = 7) starting on day 14. Scale bars: 500 μm. Collagen deposition area was calculated by affected area/ventricular area × 100%. (**C**) Reverse transcription-quantitative PCR for gene expression of collagen type I and type III in bulk heart tissues on day 56 obtained from non-EAM mice (n = 5) or from EAM mice treated daily with vehicle (n = 6), ONO-0260164 (n = 6), or both CJ-42794 and ONO-0260164 (n = 9). *P < 0.05 (vs. vehicle alone-treated EAM) or **P < 0.05 (vs. non-EAM) or ^#^*P* < 0.05 (vs. both CJ-42794 and ONO-0260164-treated EAM) calculated with 2-way ANOVA followed by the Bonferroni-Dunn post hoc testing method.

Accumulation of ECM including fibrillar collagen in the myocardial interstitium is the hallmark of cardiac fibrosis^8^ that greatly contributes to the development of DCM^19^. EAM mice on day 56 histologically had more extensive deposition of myocardial collagen, together with larger LV cavity, compared to non-EAM mice (collagen deposition area: 24.7 ± 3.0% vs. 0.7 ± 0.1%, *P* < 0.0001, respectively), while the deposition was reduced by the treatment with ONO-0260164 (collagen deposition area: 12.3 ± 2.4%, *P* = 0.0033). Moreover, this reduction was abrogated by pharmacological blockade of EP4 receptor with CJ-42794, as shown by the lack of significant difference of collagen deposition area between vehicle alone-treated EAM and both ONO-0260164 and CJ-42794-treated EAM (20.3 ± 2.7%, *P* = 0.5994, respectively) (Fig. 2A,B). There was no difference in the site of collagen deposition on the LV, such as epicardial or endocardial side, among the treatment groups (Fig. 2A).

We next evaluated cardiac gene expression of collagen type I, alpha 1 (Col1a1) and type III, alpha 1 (Col3a1) on day 56. Significantly increased their expression in the late EAM compared to non-EAM (Col1a1: 1.9 ± 0.26 vs. 1.0 ± 0.03, *P* = 0.0426; Col3a1: 3.7 ± 0.67 vs. 1.0 ± 0.02, *P* = 0.0008; respectively) was reduced by more than 50% when EAM was continuously treated with ONO-0260164 (Col1a1: 0.9 ± 0.12, *P* = 0.0259; Col3a1: 1.5 ± 0.22, *P* = 0.0046; vs. vehicle alone-treated EAM). However, the significant reduction was reversed by co-treatment with CJ-42794 (Col1a1: 2.0 ± 0.24, *P* = 0.0051; Col3a1: 3.0 ± 0.28, *P* = 0.0394; vs. ONO-0260164-treated EAM) (Fig. 2C).

Collectively, continued treatment with ONO-0260164 inhibited type I and type III collagen production and alleviated myocardial collagen deposition after myocarditis via EP4 receptor stimulation.

### The MMP-2 activation after myocarditis and its inhibition by EP4 receptor stimulant

**Figure 3:**
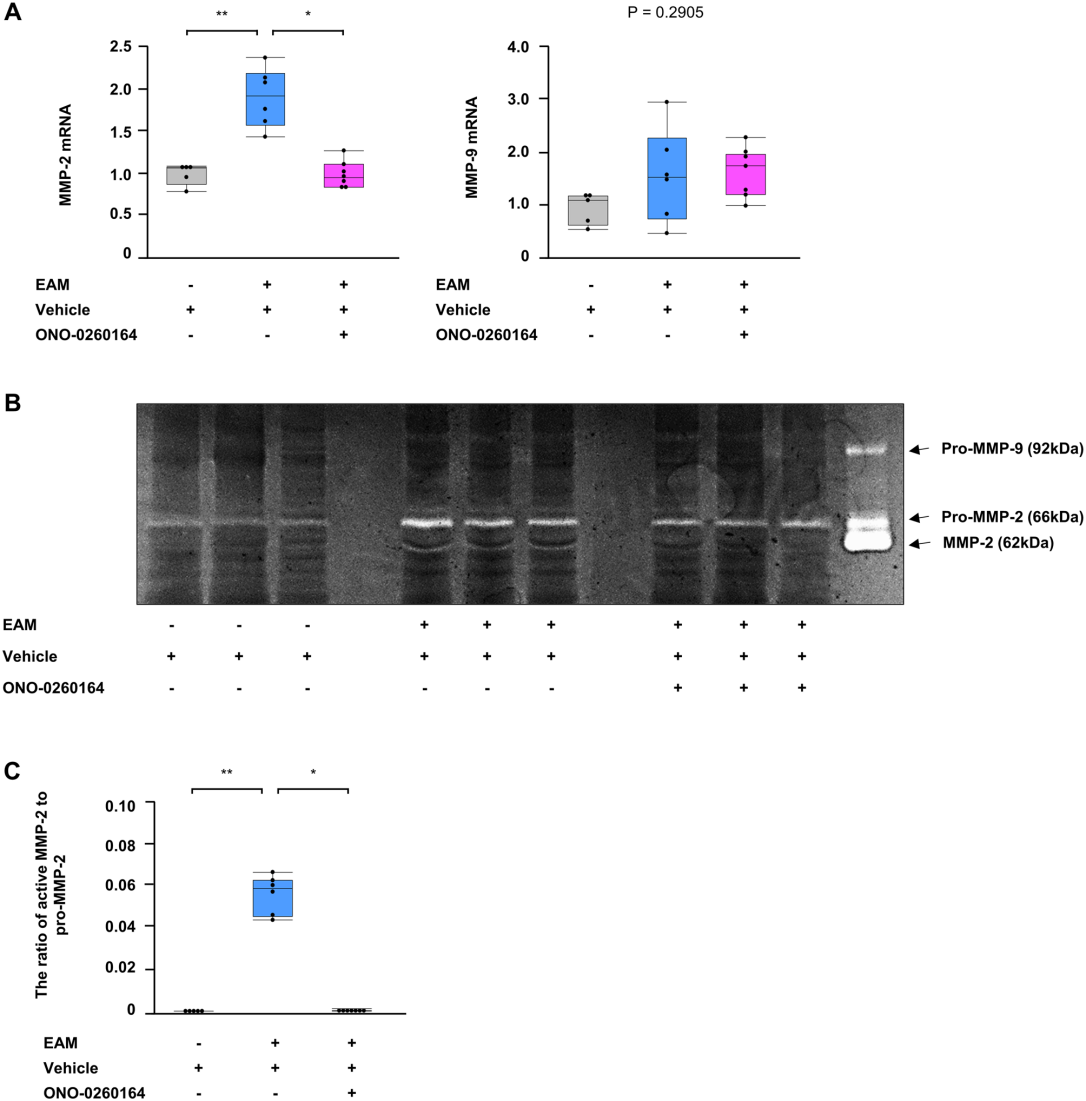
Expression and activation of matrix metalloproteinases (MMPs) in the heart of experimental autoimmune myocarditis (EAM) mice. (**A**) Reverse transcription-quantitative PCR, (**B**) representative gelatin zymography (cropped gel), and (**C**) densitometric analysis of MMPs including MMP-2 and MMP-9 in bulk heart tissues on day 56 obtained from non-EAM mice treated daily with vehicle (n = 5) or from EAM mice treated daily with vehicle (n = 6) or selective prostaglandin E2 receptor 4 (EP4) agonist (ONO-0260164) (n = 7) starting on day 14. The proteolytic bands of 62, 66, and 92 kDa corresponding to the active form of MMP-2, Pro-MMP-2, and Pro-MMP-9, respectively were scanned using a Photo scanner. Full-length gel of MMP-2 and MMP-9 is presented in Supplementary Fig. 4B. *P < 0.05 (vs. vehicle alone-treated EAM) or **P < 0.05 (vs. non-EAM) calculated with 2-way ANOVA followed by the Bonferroni-Dunn post hoc testing method.

Disruption of myocardial ECM via MMPs including MMP-2 and MMP-9 is a key trigger of adverse ventricular remodeling^20–23^, which is an important pathogenesis of DCM. To elucidate the molecular mechanisms underlying the development of DCM after myocarditis, we evaluated the expression and activation of MMP-2 and MMP-9 in the bulk heart tissues on day 56. MMP-2 gene expression was increased in EAM (EAM vs. non-EAM: 1.9 ± 0.14 vs. 1.0 ± 0.06, *P* < 0.0001, respectively), but its increase was inhibited by treatment with ONO-0260164 (1.0 ± 0.06, *P* < 0.0001 vs. vehicle alone-treated EAM). On the other hand, MMP-9 gene expression did not show any significant differences among non-EAM, vehicle-treated EAM, and ONO-0260164-treated EAM (Fig. 3A). Moreover, gelatin zymography showed that pro-MMP-2 was significantly activated in the late EAM heart compared to non-EAM heart (The ratio of active MMP-2 to pro-MMP-2: 0.0540 ± 0.0040 vs. 0.0004 ± 0.0002, *P* < 0.0001, respectively), while the activation of pro-MMP-2 was inhibited by continued treatment with ONO-0260164 (0.0003 ± 0.0002, *P* < 0.0001 vs. vehicle alone-treated EAM) (Fig. 3B,C). Active MMP-9 was not detected in the late EAM heart (Fig. 3B).

Collectively, expression and activation of MMP-2 was increased along with the DCM phenotype after myocarditis, while continued treatment with ONO-0260164 attenuated these phenomena.

### Positive regulation of TIMP-3 in the heart after myocarditis by EP4 receptor stimulant

**Figure 4:**
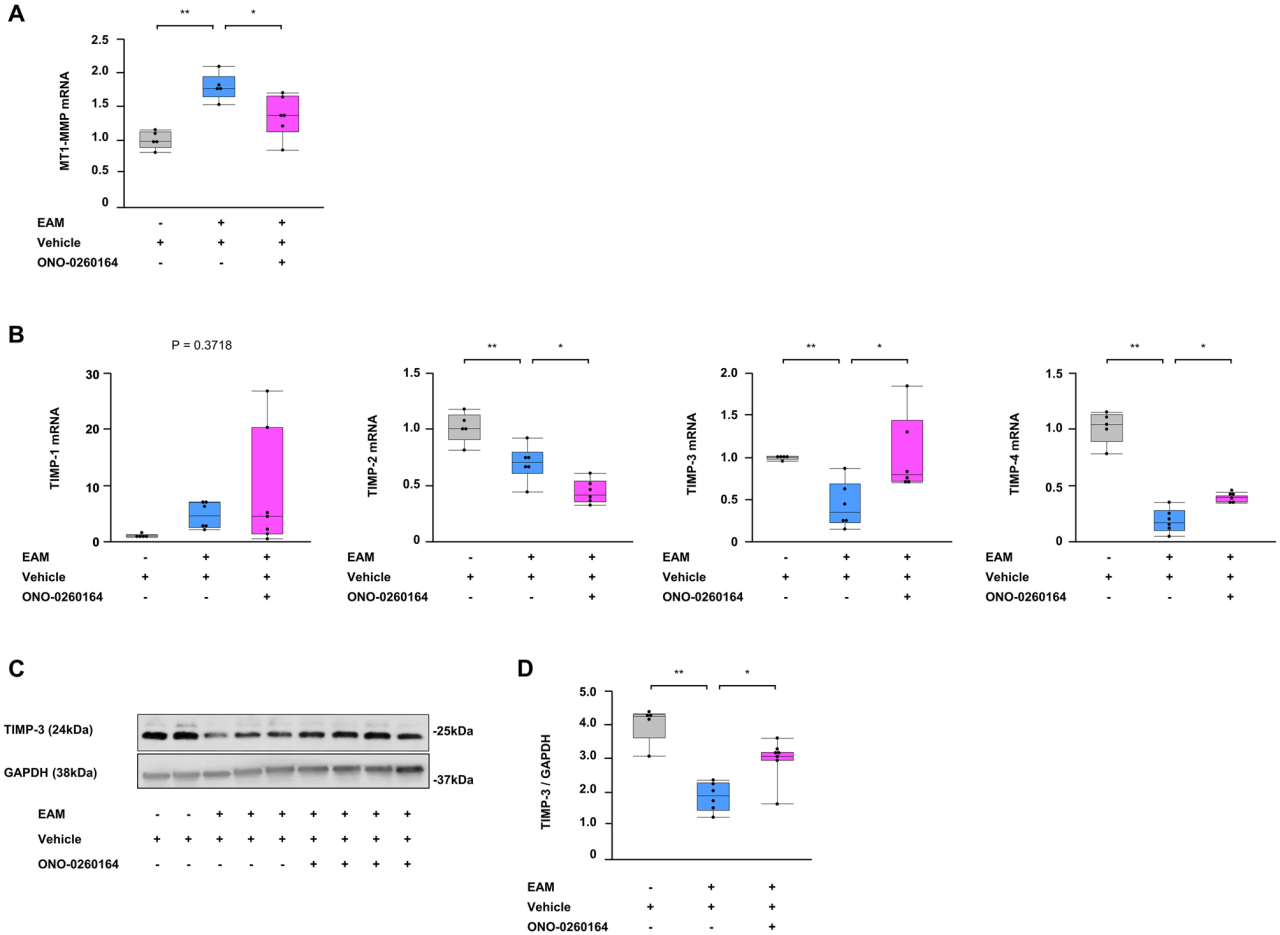
Endogenous regulators of matrix metalloproteinase (MMP) in the heart of experimental autoimmune myocarditis (EAM) mice. (**A**,**B**) Reverse transcription-quantitative PCR for membrane type (MT)-1 MMP and tissue inhibitors of metalloproteinases (TIMPs) and (**C**) representative western blot (cropped blot) and (**D**) densitometric analysis of the TIMP-3 in bulk heart tissues on day 56 obtained from non-EAM mice treated daily with vehicle (n = 5) or from EAM mice treated daily with vehicle (n = 6) or selective prostaglandin E2 receptor 4 (EP4) agonist (ONO-0260164) (n = 7) starting on day 14. Full-length blots of TIMP-3 and GAPDH are presented in Supplementary Fig. 4C. *P < 0.05 (vs. vehicle alone-treated EAM) or **P < 0.05 (vs. non-EAM) calculated with 2-way ANOVA followed by the Bonferroni-Dunn post hoc testing method.

We evaluated the molecular mechanism by which treatment with ONO-0260164 inhibits MMP-2 activation. Cardiac gene expression of membrane type 1 MMP (MT1-MMP), which is a critical endogenous activator of pro-MMP-2^24^, was significantly increased in EAM (EAM vs. non-EAM: 1.80 ± 0.09 vs. 1.01 ± 0.06, *P* = 0.0004, respectively), while its increase was significantly mitigated by treatment with ONO-0260164 (1.36 ± 0.13, *P* = 0.0231, vs. vehicle alone-treated EAM) (Fig. 4A). Moreover, among endogenous tissue inhibitors of metalloproteinases (TIMPs), which consist of four sub-types: TIMP-1, TIMP-2, TIMP-3, and TIMP-4^25^, cardiac expression of TIMP-2 gene was significantly decreased in EAM compared to non-EAM (0.69 ± 0.06 vs. 1.01 ± 0.06, *P* = 0.0042, respectively), and its expression was further reduced by treatment with ONO-0260164 (0.44 ± 0.04, *P* = 0.0133, vs. vehicle alone-treated EAM). In contrast to other TIMPs, TIMP-2 cooperates with MT1-MMP to positively regulate the activation of pro-MMP-2^24^. Cardiac gene expression of TIMP-3/4 was significantly reduced in EAM compared to non-EAM (TIMP-3: 0.45 ± 0.11 vs. 1.00 ± 0.01, *P* = 0.0293; TIMP-4: 0.19 ± 0.04 vs. 1.01 ± 0.06, *P* < 0.0001; respectively). However, their expression was increased approximately twofold by treatment with ONO-0260164 (TIMP-3: 1.03 ± 0.18, *P* = 0.0164; TIMP-4: 0.39 ± 0.01, *P* = 0.0103; vs. vehicle alone-treated EAM). Particularly, gene expression of TIMP-3 in EAM restored to an equal level to non-EAM by treatment with ONO-0260164 (non-EAM vs. ONO-0260164-treated EAM: *P* = 0.9869) (Fig. 4B). Protein expression of TIMP-3 in the heart also followed the same manner (vehicle-treated EAM vs. non-EAM: 1.82 ± 0.17 vs. 3.95 ± 0.24, *P* < 0.0001, respectively; ONO-0260164-treated EAM: 2.88 ± 0.23, *P* = 0.0069 vs. vehicle-treated EAM) (Fig. 4C,D).

Collectively, treatment with ONO-0260164 attenuated EAM-induced the reduction of TIMP-3 in the heart, contributing to the control of MMP-2 aberrant activation in EAM mice.

## Discussion

Degradation of myocardial ECM by MMPs plays an important role in the progression of ventricular remodeling^25,26^, resulting in the development of DCM. In a viral myocarditis model using BALB/c mice, MMP-3 and -9 were activated in response to inflammatory responses such as IL-1β, TNF-α, and TGF-β1, resulting in the development of inflammatory DCM with chronic myocarditis^6^. On the other hand, our study using an autoimmune myocarditis model in BALB/c mice showed that MMP-2, but not MMP-9, is activated in the pathogenesis of DCM, consistent with a previous observation in human DCM^27^. MMP-9 is inducibly expressed in neutrophils and macrophages under inflammatory conditions^28^, whereas MMP-2 is constitutively expressed in the myocardium^29^. These observations highlight that persistent activation of MMP-2 may be an important and novel molecular mechanism underlying adverse ventricular remodeling after myocarditis.

A previous report using a rat model of myocarditis have shown that EP4 receptor stimulant may be effective in the treatment of acute myocarditis^17^. However, to our knowledge, there is no report defining the therapeutic effect of EP4 receptor stimulant on the development of DCM after acute myocarditis and its molecular mechanism. Our data from the BALB/c mouse EAM model, which allows to study the transition from myocarditis to DCM phenotype^16^, showed for the first time that continued treatment with EP4 stimulant not only ameliorates cardiac malfunction with hemodynamic compromise during myocarditis, but also protects the heart affected by myocarditis from the development of DCM in association with the control of MMP-2 activation.

EP4 signaling has been shown to have dual immune functions in experimental autoimmune encephalomyelitis, facilitating Th17 cell generation in peripheral lymph node during immunization, while attenuating invasion of these cells into the brain^30,31^. We found that pharmacological blockade of the EP4 receptor increases the protein expression of RORγt, a EAM inducible Th17-specific transcription factor^18^ in the heart from EAM mice. This fact would imply that EP4 receptor stimulation inhibits the infiltration of Th17 T cells into the myocardium. Consistently, Ngoc et al. reported that EP4 stimulant suppressed T cell infiltration into myocardium and positively affected cardiac function and hemodynamics in a rat model of myocarditis^17^. This anti-inflammatory effect would contribute to the improvement of cardiac malfunction during acute myocarditis, which may result in the recovery from cardiogenic shock, a critical complication of myocarditis. Furthermore, evaluating EP4 expression in the blood vessels of EAM mice and its effect on vascular function may reveal a direct effect of EP4 stimulant on hemodynamic compromise.

Continued EP4 receptor stimulation suppressed cardiac fibrosis as well as LV remodeling in EAM mice. Inflammatory responses such as IL-1β, TNF-α, and TGF-β1 through infiltrated cells have been shown to initiate fibrotic process during myocarditis^6,25^. Thus, as shown in our and previous studies^17^, the inhibitory effect of EP4 stimulant on cell infiltration might result in alleviation of myocardial collagen deposition in EAM mice. Alternatively, digestion of myocardial ECM by MMPs also leads not only to LV remodeling but also to fibrosis through release of bioactive factors critical for fibrotic process from matrix components, including inflammatory responses such as IL-1β, TNF-α, and TGF-β1, matrikines, insulin-like growth factor, and fibroblast growth factor^25,32,33^. We found that EP4 stimulant inhibited persistent activation of MMP-2 in EAM mice. Thus, MMP-2 inactivation can be an important molecular mechanism of anti-fibrotic effect of EP4 stimulant. Our data showed that treatment with ONO-0260164 influences gene expression of MT1-MMP and TIMP-3/4, which tightly control MMP-2 activation^24,25^. Particularly, although the association of EP4 with TIMP-3 has not been elucidated, we found that the expression of TIMP-3 was decreased in the heart of EAM mice, and the decrease was restored by the administration of ONO-0260164. So far, the causal roles of MT1-MMP and TIMP-3/4 in the progression of myocardial disease have been demonstrated^34–36^. Based on these findings, myocarditis-disrupted TIMP-3-MMP-2 regulatory pathway as well as increased expression of MMP-2 may lead to the degradation of myocardial ECM that in turn promotes ventricular remodeling and stimulates new synthesis type I and III collagen^25,32^, resulting in the development of DCM, while stimulating EP4 receptor to suppress MMP-2 expression and positively regulate TIMP-3 expression may prevent DCM after myocarditis by depriving MMP-2 of its ability to degrade myocardial ECM (Supplementary Fig. 5). Our data provide a new mechanistic insight into ventricular remodeling and fibrosis after myocarditis and shed new light on the EP4 receptor as a therapeutic target for preventing DCM after myocarditis.

The rapid increase in cardiac EP4 expression during the effector phase of EAM mice from day 14 to day 21 and its persistence thereafter supported that continued treatment with ONO-0260164 from day 14 suppressed the disease progression in EAM mice. Administration of EP4 stimulant in patients with acute myocarditis may be therapeutically effective to prevent not only the progression to fulminant case characterized by cardiogenic shock but also the subsequent development of DCM.

In a previous study using cultured human aortic smooth muscle cells or human abdominal aortic aneurism tissue organ cultures containing smooth muscle cells and macrophages, EP4 stimulation with ONO-AE1-329 increased MMP-2 activation, while ONO-AE3-208, an EP4 antagonist decreased its activation^37^. These observations were contrast to our results. However, in another study using cardiac fibroblast, EP4 agonist reduced the activation of MMP-2 and the expression of its key activator, MT1-MMP^38^. Moreover, aged mice lacking the EP4 receptor in cardiomyocytes display a phenotype of dilated cardiomyopathy coupled with increased expression of MMP-2 as well as MT1-MMP in the left ventricle^39^. Stimulation of EP4 receptor may produce different phenotypes on different types of cells and organs.

Long-term administration of a high dose of EP4 selective agonist might lead to cardiac hypertrophy and dyslipidemia. In our in vivo experimental setting with healthy mice, continued administration of 50 mg/kg/day of ONO-0260164 for 49 days significantly increased LV mass index via increasing LV wall thickness rather than LV dimensions. Consistently, several reports have demonstrated that activation of EP4 receptor signaling contributes to the PGE2-mediated cardiac hypertrophy, which is characterized by larger cross-sectional area of cardiomyocytes^40,41^. Moreover, in our study, continued administration of the high dose also increased body weight, consistent with a previous report showing that animal models mice lacking EP4 exhibited slower weight gain and reduced adiposity upon high fat diet challenge when compared with wild type mice^42^. Since activation of PGE2-EP4 signaling can exert multiple biochemical effects including cardiac function and structure and lipid metabolism, setting the appropriate therapeutic dose for the diseases is very important.

In conclusion, the present study using EAM mice delineated for the first time that myocardial ECM metabolism by persistent activation of MMP-2 is an important and novel molecular mechanism underlying adverse ventricular remodeling after myocarditis and that continued treatment with selective EP4 receptor stimulant rescues cardiac malfunction during myocarditis and exhibits robust preventive effects against adverse ventricular remodeling and cardiac fibrosis after myocarditis in association with the TIMP-3-MMP-2 regulatory pathway. EP4 receptor stimulant could potentially become a part of new strategy to prevent heart failure and DCM caused by inflammatory heart disease.

## Methods

### Ethics statement

All animal studies were approved by the Institutional Animal Care and Use Committees of Yokohama City University (license number: F-A-18-043) and carried out in accordance with the guidelines of Yokohama City University. Moreover, the present study was also carried out in compliance with the ARRIVE (Animal Research: Reporting of In Vivo Experiments) guidelines (https://arriveguidelines.org/arrive-guidelines).

### Induction of EAM model

Six- to eight-week-old male BALB/cJ background mice (Jackson Laboratory) were subcutaneously immunized with 150 µg of myocarditogenic peptide (Alpha-Myosin Heavy Chain-MyHC-α_614–634_, Ac-SLKLMATLFSTYASAD-OH, AnaSpec, Belgium) emulsified 1:1 in PBS/complete Freund’s adjuvant (CFA) (1 mg/ml, H37Ra; Difco) or PBS/CFA only on days 0 and 7^43^. Before immunization, all mice were anesthetized with 2.0 vol% isoflurane inhalation in oxygen. Myocarditis severity was evaluated by using a 0–4 scoring system^43^.

### Treatment protocols

A EP4 selective agonist, ONO-0260164 was kindly provided by Ono Pharmaceutical Co., Ltd (Osaka, Japan)^15^. A EP4 selective antagonist, CJ-42794 was purchased from Cayman Chemicals (Ann Arbor, MI, USA).

At 2 weeks after immunization with myosin/CFA/PBS, the EAM mice were randomly separated into subgroups and daily administered 20 mg/kg of ONO-0260164, 30 mg/kg of CJ-42794, or vehicle via gastric gavage from day 14 to day 21 and 56 according to treatment protocols as follows: (A) Either CJ-42794 or vehicle (0.5% methylcellulose) for 1 week (day 14–21); (B) Either ONO-0260164 or vehicle (water) for 1 or 6 weeks (day 14–21 or day 14–56); (C) both ONO-0260164 and CJ-42794, ONO-0260164 only, or vehicle only (water/0.5% methylcellulose) for 1 or 6 weeks (day 14–21 or day 14–56). On the other hand, non-EAM mice were treated daily with vehicle only starting 2 weeks after immunization with CFA/PBS alone (day 14–21 or day 14–56). Apart from these protocols, to clarify the dose-dependent in vivo responses of normal mice to the drug, water, 2, 20, or 50 mg/kg of ONO-0260164 was administered to unimmunized healthy mice.

### Echocardiographic measurements

Trans-thoracic echocardiography was performed with a Vevo 3100LT system (VisualSonics, Fujifilm, Tokyo, Japan) equipped with a MX400 20–46 MHz linear array transducer (Visual Sonics, Fujifilm, Tokyo, Japan) at 14, 21, and 56 days after immunization. The mice were anesthetized by 2.0 vol% isoflurane inhalation in oxygen and were placed in a lateral position on a heating pad and chest hair was removed. Heart rate was monitored continuously during the examination. Hearts were imaged in the two-dimensional mode in short-axis views at the level of papillary muscle. M-mode views were used to measure the left ventricular (LV) dimensions according to the American Society for Echocardiography leading edge method^44^, including LV end-diastolic dimension (LVDd), LV end-systolic dimension (LVDs), interventricular septum diastolic or systolic thickness (IVSd or IVSs), and LV posterior wall diastolic or systolic thickness (PWd or PWs). Fractional shortening (FS = [(LVDd − LVDs)/LVDd] × 100) and LV mass index (LVMI) [LV mass/body weight] = 1.04 [(LVDd + PWd + IVSd)^3^ − LVDd^3^] × 0.8 + 0.6/g) were calculated with Vevolab software (VisualSonics version 3.2.0, Fujifilm, Tokyo, Japan). All echocardiographic examinations were performed by the same examiner blinded to the identity of the mouse.

### Blood pressure measurement

Systolic blood pressure (BP) and heart rate (HR) were evaluated before and 1 and 2 h after a single administration or after daily administration for 0, 21, or 49 days in healthy mice or evaluated at day 21 or 56 after immunization in EAM or non-EAM mice. The BP and HR were measured at the same time point in conscious mice by using a tail-cuff system (MK-2000; Muromachi Kikai Co., Tokyo, Japan). All examinations were performed by the same examiner blinded to the identity of the mouse.

### Isolation and preservation of heart samples

Anesthetized mice were perfused with cold PBS until the liver turned whitish after blood (> 700 µL) was collected. Subsequently, hearts were removed from the euthanized mice, and the isolated ventricles were stored at − 80 °C until protein and mRNA analyses. Alternatively, isolated ventricles for myocardial pathology were cut at the level of the papillary muscles and then harvested in 10% formalin solution. Anesthesia and euthanasia for all mouse experiments were performed by continuous inhalation of 5.0 vol% isoflurane.

### Pathology

We obtained transverse sections of paraffin-embedded heart (5 µm) for histopathological examination. To identify myocardial inflammation and collagen deposition, ventricular cross-sections, which were deparaffinized and subsequently rehydrated, were then stained with hematoxylin–eosin and picrosirius red, respectively. Collagen staining was performed according to manufacturer protocol (Picro-Sirius Red Stain Kit [For Cardiac Muscle], SRC-1, ScyTek Lab). Area of myocardial inflammation or collagen deposition was calculated as the area ratio (affected area/LV area × 100%) using the Image J software. Values for three ventricular regions were averaged for each heart, and the mean percentage of affected area for each group was calculated. All data were analyzed in a blind fashion by two independent investigators and averaged.

### Immunohistochemistry

Ventricular cross-sections of paraffin-embedded heart tissue, which were removed 21 days after immunization with cardiac myosin, were deparaffinized and rehydrated, and then endogenous peroxidase activity was blocked in 3% hydrogen peroxide in methanol for 10 min. After blocking with 2.5% BSA in PBS for 30 min, samples were incubated overnight at 4 °C with primary anti-body for EP4 (1:100) (Santa Cruz Biotechnology, CA, USA) and then incubated at room temperature with the VECTASTAIN ABC Kit according to manufacturer protocol (Vector laboratories, CA, USA). Ultimately, EP4 signal was detected with streptavidin-peroxidase complex with diaminobenzidine. Negative control sections were incubated with secondary Ab alone.

### RNA extraction, reverse transcription, and quantitative real-time PCR

RNA was isolated from heart tissues with Trizol Reagent (Invitrogen) according to the manufacturer’s protocol. cDNA synthesis was performed using 2.0 μg RNA and PrimeScript RT reagent Kit (Takara, Tokyo, Japan). Quantitative real-time PCR was performed with TB Green Premix Ex Taq II (Takara, Tokyo, Japan) using a CFX96 Real-Time PCR Detection System (Bio-Rad), with GAPDH as internal control. Reactions were performed in 25-μL containing cDNA (2.0 μL), TB Green Premix Ex Taq II (2×, 12.5 μL), PCR forward Primer (10 μM, 1.0 μL), PCR reverse primer (10 μM, 1.0 μL), and ddH2O (8.5 μL). PCR was performed at 95 °C for 30 s, followed by 50 cycles of 95 °C for 5 s and 60 °C for 30 s. Gene expression was analyzed using the 2^−ΔΔCt^ method. Target mRNA expression was compared as relative ratio to the housekeeping gene GAPDH. The sequences of the primers are shown in Supplementary Table 2.

### Western blot analysis

Cardiac tissue homogenates were lysed in NP-40 with protease inhibitor. Proteins (25 μg/well) were separated by SDS-PAGE, transferred to a nitrocellulose membrane, and incubated with primary antibodies to EP4 (Santa Cruz Biotechnology, CA, USA), tissue inhibitor of metalloproteinase (TIMP)-3 (Proteintech, IL, USA), and GAPDH (Cell Signaling Technology, MA, USA) at 4 °C overnight. The membranes were then incubated with a secondary antibody (Cell Signaling Technology, MA, USA) for 1 h at room temperature and developed with ECL reagent. Enhanced chemiluminescence was detected with an LAS-3000 (Fujifilm, Tokyo, Japan).

### Gelatin zymography

Proteolytic activity of cardiac tissue homogenates was examined by gelatin zymography according to the manufacturer’s protocol (Cosmo Bio Co., Ltd., Tokyo, Japan). Samples (25 μg/well) were subjected to 10% SDS–polyacrylamide gel electrophoresis using gels containing 0.1% gelatin. Gels were incubated at 37 °C for 24 h in enzyme reaction buffer and then stained with 0.5% Coomassie Brilliant Blue R-250 in 50% methanol and 10% acetic acid for 1 h. MMP marker (Cosmo Bio Co., Ltd.) was used as a matrix metalloproteinase marker. The proteolytic bands of 62, 66, and 92 kDa corresponding to the active form of MMP-2, Pro-MMP-2, and Pro-MMP-9, respectively were scanned using a Photo scanner.

### Statistical analysis

Comparisons between the 2 groups with the different treatment were performed by the nonparametric 2-tailed Mann–Whitney test. Data from different time points or different treatments were analyzed with 2-way ANOVA followed by the Bonferroni-Dunn post hoc testing method. All experiments were performed independently at least three times. All data were analyzed in a blind fashion by two independent investigators and averaged. All data are presented as dot and box plots or mean ± SEM [95% confidence interval]. A *P* value less than 0.05 was considered significant. Data analyses were done using the JMP ver. 12.2 software.

## Supplementary Information

Supplementary Information 1.

Supplementary Video 1.

Supplementary Video 2.
