# Supplementary material for: Prostaglandin-E2 receptor-4 stimulant rescues cardiac malfunction during myocarditis and protects the heart from adverse ventricular remodeling after myocarditis
Source: Sci Rep. 2021 Oct 26;11:20961. doi: 10.1038/s41598-021-99930-5 (PMC8548292; doi:10.1038/s41598-021-99930-5)
Supplement: Supplementary file 1 — Supplementary Information 1. [file 41598_2021_99930_MOESM1_ESM.pdf]

Supplemental material

Prostaglandin-E2 Receptor-4 Stimulant Rescues Cardiac Malfunction during Myocarditis and Protects the Heart from Adverse Ventricular Remodeling after Myocarditis

Akira Takakuma, Mototsugu Nishii, Alan Valaperti, Haruto Hirag, Ryo Saji, Kazuya Sakai, Reo Matsumura, Yasuo Miyata, Nozomu Oba, Fumiya Nunose, Fumihiro Ogawa, Kouichi Tamura, Ichiro Takeuchi

Content

Supplementary Figures and legends: 1-5

Supplementary Tables: 1-2

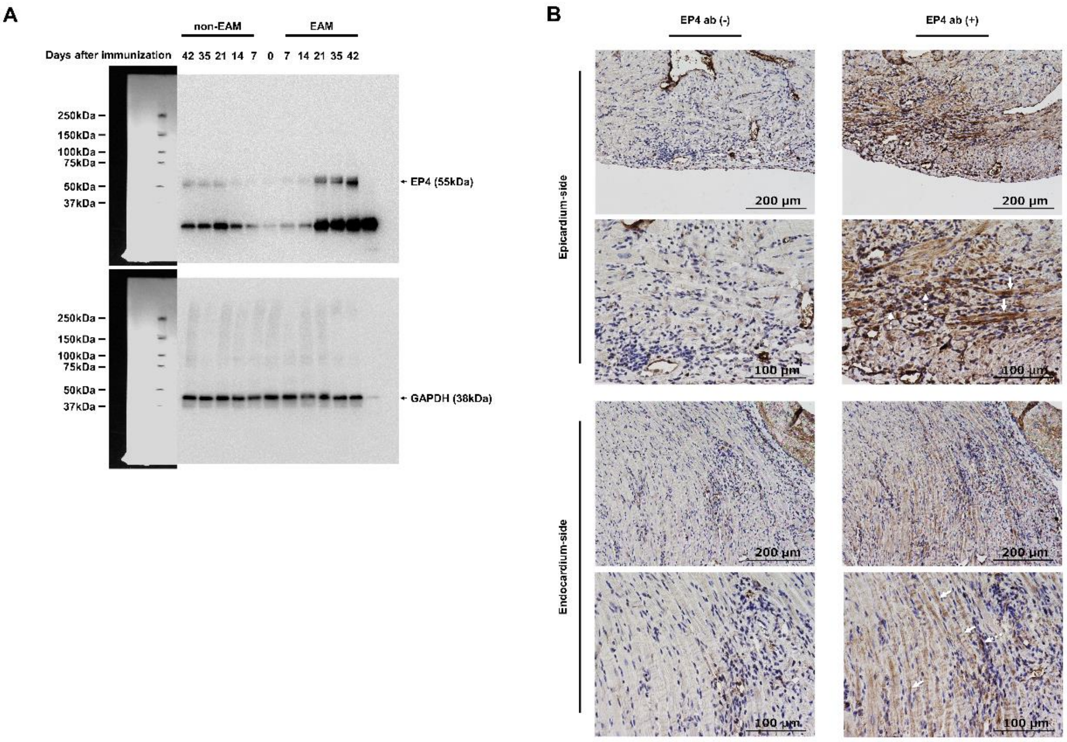

Supplementary Figure 1. Prostaglandin-E2 receptor-4 (EP4) expression in the experimental autoimmune myocarditis (EAM).

(**A**) Representative full-length western blots of EP4 as well as GAPDH in bulk heart tissues over time after the immunization with PBS / complete Freund's adjuvant (CFA) (non-EAM, left panel) or cardiac myosin / CFA (EAM, right panel). (**B**) Representative immunohistochemistry staining of EP4 in ventricular cross-sections obtained from EAM mice on day 21. Scale bars: 100 and 200 μm. EP4 ab (-): staining with only secondary antibody.

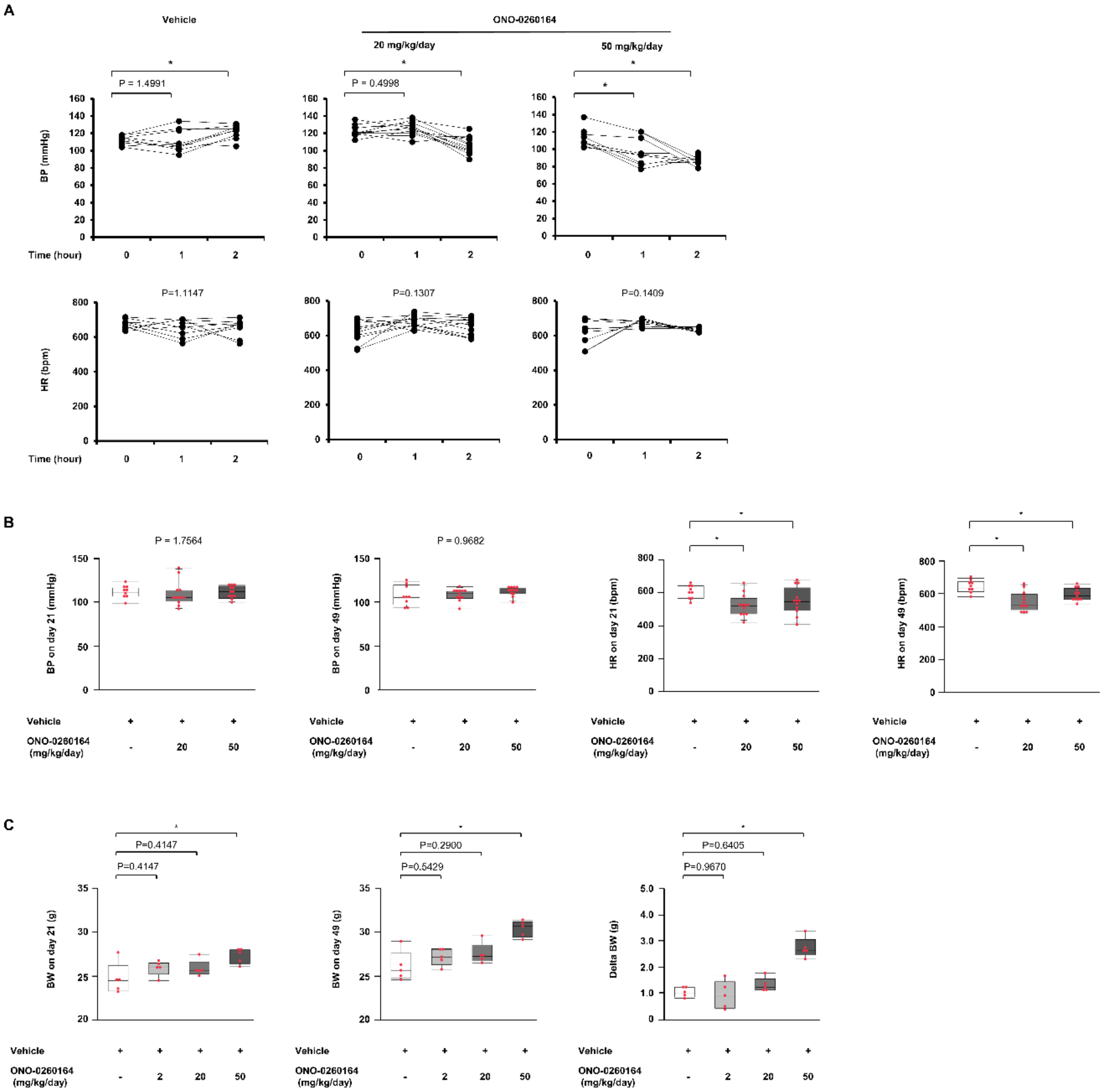

Supplementary Figure 2. Effects of ONO-0260164, a selective prostaglandin-E2 receptor-4 (EP4) agonist on healthy mice.

(**A**) Systolic blood pressure (BP) and heart rate (HR) after a single administration of vehicle (n=9) or 20 mg/kg (n=12) or 50 mg/kg (n=9) of ONO-0260164. The BP was significantly increased 2 hours after a single administration of vehicle compared with before administration (121 ± 2.7 mmHg vs. 112 ± 1.7 mmHg, P = 0.0009, respectively). Conversely, BP was decreased 1 or 2 hours after a single administration of ONO-0260164 (20 or 50

mg/kg) compared with before the administration (20 mg/kg: [2 hours] 106 ± 2.9 mmHg vs. 123 ± 1.8 mmHg, P < 0.0001; 50 mg/kg: [1 hour] 98 ± 4.7 mmHg vs. 113 ± 3.1 mmHg, P = 0.0009, [2 hours] 87 ± 1.5 mmHg vs. 113 ± 3.1 mmHg, P < 0.0001; respectively), while the HR did not change among before and 1 and 2 hours after the administration of vehicle or ONO-0260164 (vehicle: 673 ± 8 bpm vs. 653 ± 15 bpm vs. 654 ± 14 bpm, ANOVA P=1.1147; 20 mg/kg of ONO-0260164: 630 ± 18 bpm vs. 678 ± 10 bpm vs. 653 ± 15 bpm, ANOVA P=0.1307; 50 mg/kg of ONO-0260164: 619 ± 22 bpm vs. 673 ± 6 bpm vs. 633 ± 3 bpm, ANOVA P=0.1409; respectively). (**B**) BP and HR in healthy mice treated daily with vehicle alone (n=9) or 20 mg/kg (n=12) or 50 mg/kg (n=12) of ONO-0260164 daily. ONO-0260164 did not affect BP on days 21 and 49 (vehicle alone vs. 20 mg/kg/day vs. 50 mg/kg/day: [day 21] 114 ± 2.2 mmHg vs. 112 ± 3.9 mmHg vs. 114 ± 1.9 mmHg, ANOVA P=1.7564; [day 49] 110 ± 3.5 mmHg vs. 111 ± 1.8 mmHg vs. 114 ± 1.3 mmHg, ANOVA P=0.9682; respectively). However, the HR was significantly reduced in 20 mg/kg/day or 50 mg/kg/day of ONO-0260164 compared with in vehicle alone (day 21: 541 ± 18 bpm or 564 ± 23 bpm vs. 614 ± 12 bpm, P = 0.0021 or P = 0.0330; day 49: 568 ± 16 bpm or 609 ± 10 bpm vs. 652 ± 11 bpm, P < 0.0001 or P = 0.0068; respectively). (**C**) Body weight (BW) in healthy mice treated daily with vehicle alone (n=5) or with 2 mg/kg (n=5), 20 mg/kg (n=5), or 50 mg/kg (n=5) of ONO-0260164 daily. The BWs on days 21 and 49 were significantly increased in the 50 mg/kg/day group of ONO-0260164 compared to the vehicle only group (day 21: 27.8 ± 0.4 g vs. 25.4 ±

0.8 g, P = 0.0138; day 49: 30.7 ± 0.4 g vs. 26.7 ± 0.7 g, P = 0.0003; respectively), but not in the 2 or 20 mg/kg/day group (day 21: 26.5 ± 0.3 g, P = 0.4147 or 26.5 ± 0.4 g, P = 0.4147 vs. the vehicle only group; day 49: 27.7 ± 0.4 g, P = 0.5429 or 28.1 ± 0.5 g, P = 0.2900 vs. the vehicle only group; respectively). Furthermore, the change in BW from day 21 to day 49 was significantly increased in the 50 mg/kg/day group of ONO-0260164 compared to the vehicle only group (2.9 ± 0.2 g vs. 1.3 ± 0.1 g, P < 0.0001; respectively), but not in the 2 or 20 mg/kg/day groups of ONO-0260164 (1.2 ± 0.2 g, P = 0.9670; 1.6 ± 0.1 g, P = 0.6405; vs. the vehicle only group; respectively). P* < 0.05 (vs. before administration or vehicle alone). Data from different time points or different treatments were analyzed with 2-way ANOVA followed by the Bonferroni-Dunn post hoc testing method.

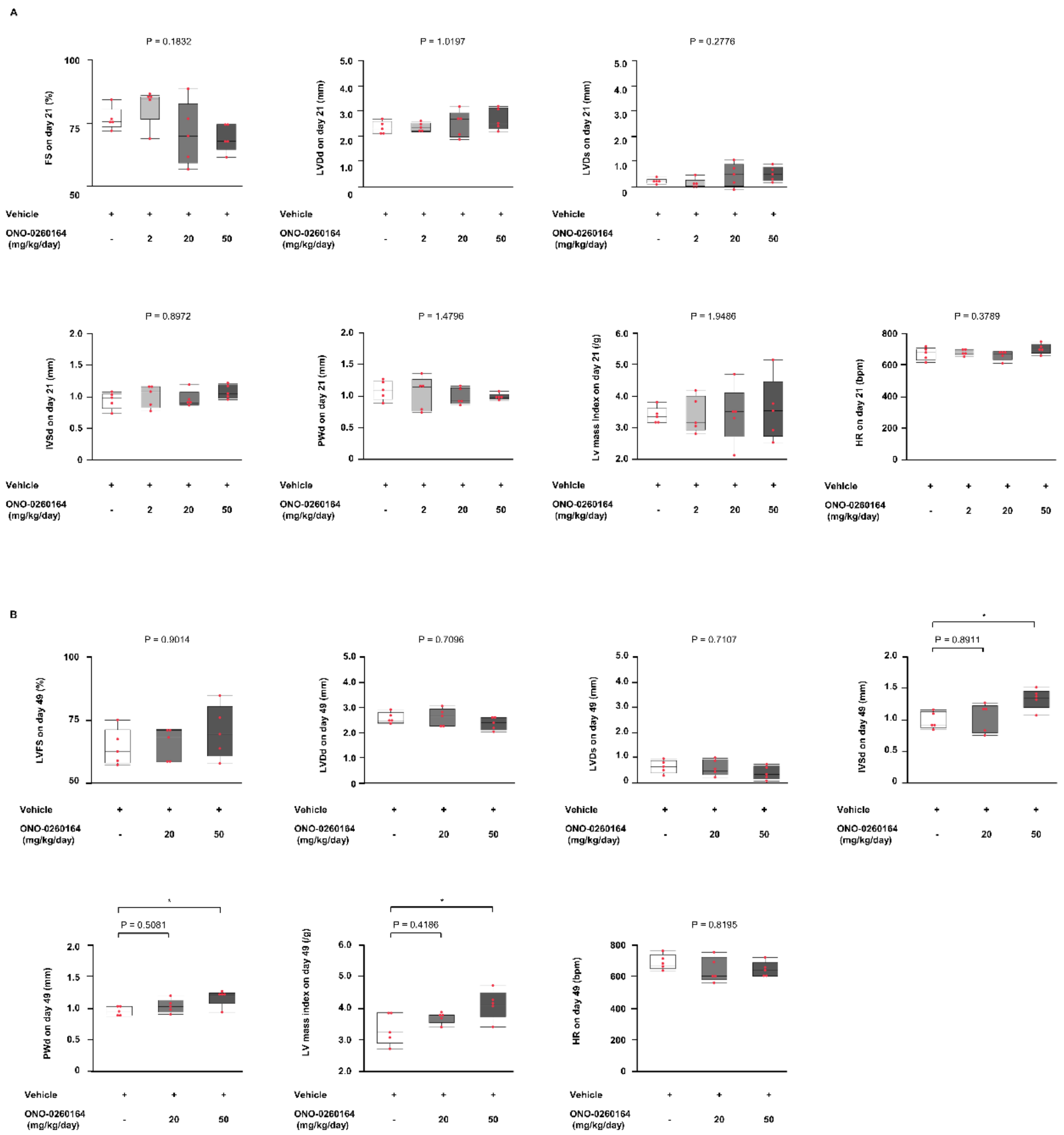

Supplementary Figure 3. Echocardiography in healthy mice treated daily with ONO-0260164, a selective prostaglandin-E2 receptor-4 (EP4) agonist.

(**A**) Echocardiographic findings on day 21 in healthy mice treated with vehicle alone or with 2 mg/kg, 20 mg/kg, or 50 mg/kg of ONO-0260164 (n=5 / each). Left ventricular fractional shortening (LVFS), LV end-systolic dimension (LVDd), LV end-systolic dimension (LVDs), LV posterior wall diastolic thickness (PWd), interventricular septum diastolic thickness (IVSd),

and LV mass index (LVMI) did not show any significant differences among vehicle, the 2 mg/kg/day group, the 20 mg/kg/day group, and the 50 mg/kg/day group (LVFS: 78.5 ± 1.9 % vs. 83.2 ± 3.0 % vs. 72.9 ± 5.3 % vs. 71.4 ± 2.3 %, ANOVA P = 0.1832; LVDd: 2.5 ± 0.1 mm vs. 2.5 ± 0.1 mm vs. 2.6 ± 0.2 mm vs. 2.8 ± 0.2 mm, ANOVA P = 1.0197; LVDs: 0.53 ± 0.04 mm vs. 0.43 ± 0.08 mm vs. 0.76 ± 0.19 mm vs. 0.80 ± 0.12 mm, ANOVA P = 0.2776; IVSd: 1.0 ± 0.06 mm vs. 1.1 ± 0.07 mm vs. 1.0 ± 0.06 mm vs. 1.1 ± 0.05 mm, ANOVA P = 0.8972; PWd: 1.2 ± 0.06 mm vs. 1.1 ± 0.11 mm vs. 1.1 ± 0.06 mm vs. 1.1 ± 0.02 mm, ANOVA P = 1.4796; LVMI: 3.6 ± 0.1/g vs. 3.6 ± 0.2/g vs. 3.6 ± 0.4/g vs. 3.7 ± 0.4/g, ANOVA P = 1.9486; HR: 676 ± 16 bpm vs. 685 ± 8 bpm vs. 669 ± 13 bpm vs. 708 ± 14 bpm, ANOVA P = 0.3789; respectively). (**B**) Echocardiographic findings on day 49 in healthy mice treated with vehicle alone or with 20 mg/kg or 50 mg/kg of ONO-0260164 (n=5 / each). LVFS, LVDd, LVDs, and HR did not show any significant differences among vehicle alone, 20 mg/kg/day, and 50 mg/kg/day (LVFS: 66.4 ± 3.0 % vs. 67.6 ± 2.7 % vs. 72.2 ± 4.4 %, ANOVA P = 0.9014; LVDd: 2.7 ± 0.1 mm vs. 2.8 ± 0.1 mm vs. 2.5 ± 0.1 mm, ANOVA P = 0.7096; LVDs: 0.9 ± 0.1 mm vs. 0.9 ± 0.1 mm vs. 0.7 ± 0.1 mm, ANOVA P = 0.7107; HR: 697 ± 21 bpm vs. 652 ± 33 bpm vs. 656 ± 20 bpm, ANOVA P = 0.8195; respectively). However, IVSd, PWd, and LVMI were significantly increased in 50 mg/kg/day group of ONO-0260164 compared with in vehicle only group (IVSd; 1.4 ± 0.07 mm vs. 1.1 ± 0.06 mm, P = 0.0277; PWd: 1.2 ± 0.05 mm vs. 1.0 ± 0.03 mm, P = 0.0212; LVMI: 4.2 ± 0.2/g vs. 3.5 ± 0.2/g, P = 0.0282; respectively). These

parameters did not show any significant differences between 20 mg/kg/day and vehicle alone (IVSd: 1.1 ± 0.1 mm, P = 0.8911; PWd: 1.1 ± 0.05 mm, P = 0.5081; LVMI: 3.8 ± 0.1/g, P = 0.4186; respectively). P* < 0.05 (vs. vehicle alone). Data from different treatments were analyzed with 2-way ANOVA followed by the Bonferroni-Dunn post hoc testing method.

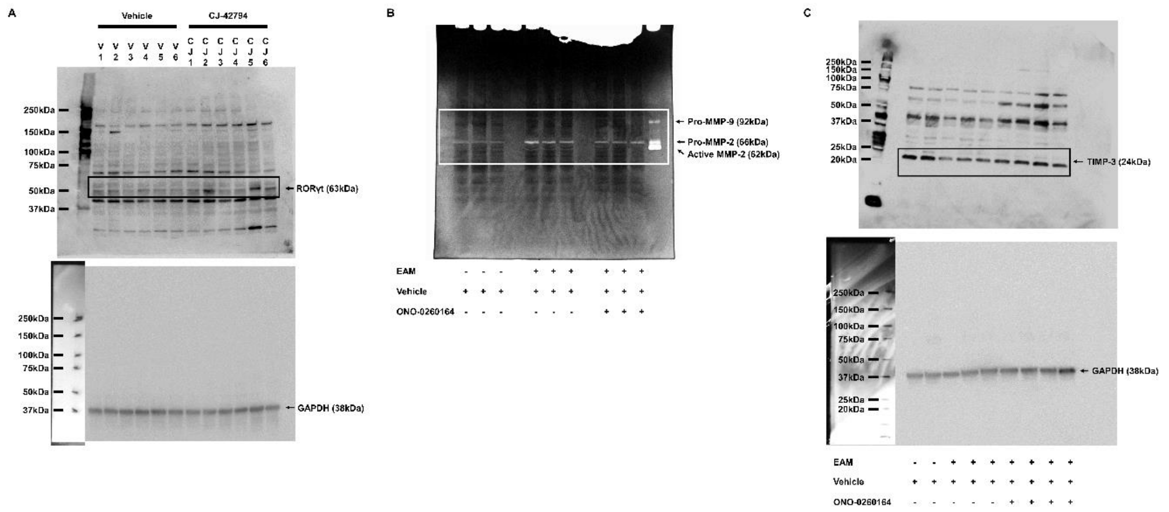

Supplementary Figure 4. Full-length western blots and gel.

(**A**) Western blots of the EAM inducible Th17-specific master transcription factor, retinoic acid receptor-related orphan nuclear receptor (ROR γt) and GAPDH with protein markers. (**B**) Gelatin zymography gel of matrix metalloproteinase (MMP)-2 and -9 with the proteolytic bands of 62, 66, and 92kDa corresponding to the active form of MMP-2, Pro-MMP-2, and Pro-MMP-9, respectively. (**C**) Western blots of the tissue inhibitor of metalloproteinase (TIMP)-3 and GAPDH with protein markers.

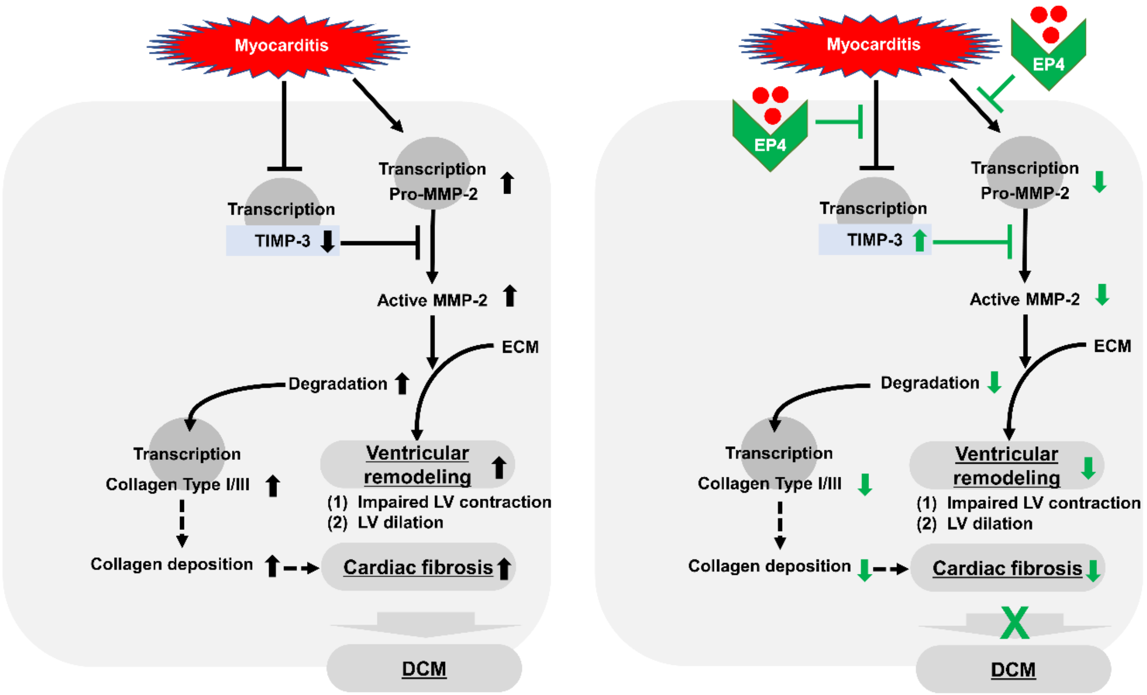

Supplementary Figure 5. Schematic model illustrating the molecular mechanism for regulation of ventricular remodeling after myocarditis by prostaglandin E2 receptor 4 (EP4) stimulant.

(A) schema for experimental autoimmune myocarditis (EAM). (B) schema for treatment with EP4 stimulant. MMP: matrix metalloproteinase; TIMP: tissue inhibitor of metalloproteinase; ECM: extracellular matrix; DCM; dilated cardiomyopathy; Red circles: ONO-0260164, a selective EP4 stimulant.

Supplementary Table 1. Echocardiographic data on day 14 in experimental autoimmune myocarditis (EAM) mice

	non-EAM (n=10)	EAM (n=15)	P value
Parameters			
LVFS (%)	72.7 ± 1.4 [69.5-75.8]	50.0 ± 3.9 [41.7-58.3]	< 0.0001
LVDs (mm)	0.71 ± 0.02 [0.66-0.77]	1.43 ± 0.20 [1.00-1.87]	< 0.0001
LVDd (mm)	2.52 ± 0.02 [2.47-2.57]	2.69 ± 0.16 [2.35-3.04]	0.8027
HR (bpm)	690.1 ± 13.4 [659.8-720.4]	644.4 ± 15.1 [612.2-676.7]	0.0345
BP (mmHg)	118.9 ± 1.3 [115.8-121.9]	90.3 ± 1.6 [87.0-93.6]	0.0004

Data are mean±SEM [95% confidence interval]. LVFS: Left ventricular fractional shortening; LVDs: LV end-systolic dimension; LVDd: LV end-diastolic dimension; HR: Heart rate; BP; systolic blood pressure. P values were calculated with nonparametric 2-tailed Mann-Whitney *U* test.

Supplementary Table 2. Sequences of Primers used for RT-PCR

Gene	Sense	Antisense
Col1a1	CCTCAGGGTATTGCTGGACAAC	CAGAAGGACCTTGTTTGCCAGG
Col3a1	GACCAAAAGGTGATGCTGGACAG	CAAGACCTCGTGCTCCAGTTAG
MMP-2	ACGATGATGACCGGAAGT	GTGTAGATCGGGGCCATC
MMP-9	CAGACGTGGGTCGATTCC	TCATCGATCATGTCTCGC
MT1-MMP	CCCTAGGCCTGGAACATTCT	TTTGGGCTTATCTGGGACAG
TIMP-1	CCCAGAAATCAACGAGA	TGGGACTTGTGGGCATA
TIMP-2	ACGCTTAGCATCACCCAGAAG	TGGGACAGCGAGTGATCTTG
TIMP-3	ACACGGAAGCCTCTGAAA	TGGAGGTCACAAAACAAGG
TIMP-4	CACCCTCAGCAGCACATCTG	GGCCGGAACTACCTTCTCACT
GAPDH	ACTCCACTCACGGCAAATTCA	GGTCTCGCTCCTGGAAGATG

Col1a1: collagen type I, alpha 1, Col3a1: collagen type III, alpha 1, MMP: matrix metalloproteinase, MT1-MMP: membrane type 1 MMP, TIMP: tissue inhibitors of metalloproteinase.
