# Supplementary material for: Prostaglandin-E2 receptor-4 stimulant rescues cardiac malfunction during myocarditis and protects the heart from adverse ventricular remodeling after myocarditis
Source: Sci Rep. 2021 Oct 26;11:20961. doi: 10.1038/s41598-021-99930-5 (PMC8548292; doi:10.1038/s41598-021-99930-5)
Supplement: Supplementary file 2 — Supplementary Video 1. [file 41598_2021_99930_MOESM2_ESM.docx]

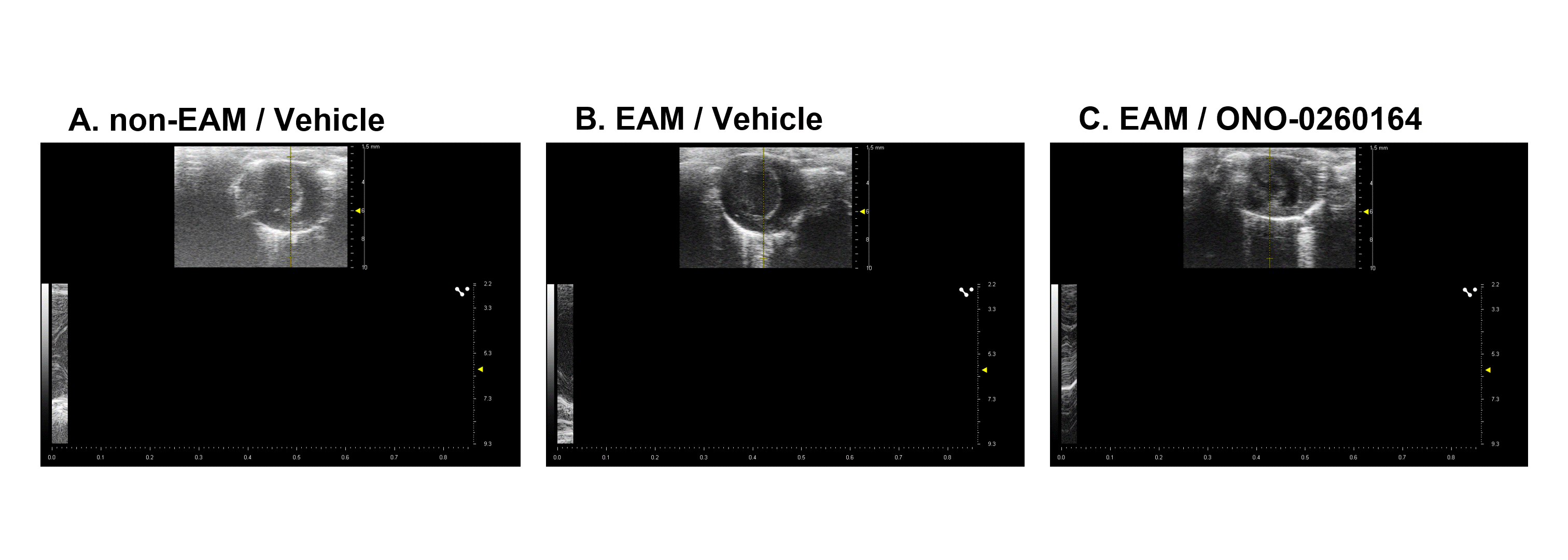
**Supplementary Video 1.** **Representative echocardiography on day 21 in experimental autoimmune myocarditis (EAM) mice.**

(**A**) non-EAM mice treated with vehicle alone; (**B**) EAM mice treated with vehicle alone; (**C**) EAM mice treated daily with ONO-0260164, a selective prostaglandin-E2 receptor-4 (EP4) agonist.
